# Inflammation-linked adaptations in dermal microvascular reactivity accompany the development of obesity and type 2 diabetes

**DOI:** 10.1038/s41366-018-0148-4

**Published:** 2018-07-13

**Authors:** Marie-Sophie Nguyen-Tu, Pierre Nivoit, Valérie Oréa, Sandrine Lemoine, Cécile Acquaviva, Aurélie Pagnon-Minot, Bérengère Fromy, Jaswinder K. Sethi, Dominique Sigaudo-Roussel

**Affiliations:** 1 LBTI, UMR CNRS 5305, 69367 Lyon Cedex 07, France; 2 University of Lyon 1, 69367 Lyon Cedex 07, France; 3 Centre de Biologie et Pathologie Est, University Hospital, Hospices Civils de Lyon, 69677 Bron, France; 4 Novotec, ZAC du Chêne Europarc, 11 rue Edison, 69500 Bron, France; 5 Faculty of Medicine, University of Southampton, Institute of Developmental Sciences Building, Southampton General Hospital, Southampton, SO16 6YD UK; 6 National Institute for Health Research Southampton Biomedical Research Centre, University Hospital Southampton NHS Foundation Trust, Southampton General Hospital, Southampton, SO16 6YD UK; 7 Institute for Life Sciences, Life Sciences Building 85, University of Southampton, Highfield, Southampton, SO17 1BJ UK

**Keywords:** Obesity, Obesity

## Abstract

**Background/Objectives:**

The increased prevalence of obesity has prompted great strides in our understanding of specific adipose depots and their involvement in cardio-metabolic health. However, the impact of obesity on dermal white adipose tissue (dWAT) and dermal microvascular functionality remains unclear. This study aimed to investigate the temporal changes that occur in dWAT and dermal microvascular functionality during the development of diet-induced obesity and type 2 diabetes in mice.

**Methods:**

Metabolic phenotyping of a murine model of hypercaloric diet (HCD)-induced obesity and type 2 diabetes was performed at three time points that reflected three distinct stages of disease development; 2 weeks of HCD-overweight-metabolically healthy, 4 weeks of HCD-obese-prediabetic and 12 weeks of HCD-obese-type 2 diabetic mice. Expansion of dWAT was characterized histologically, and changes in dermal microvascular reactivity were assessed in response to pressure and the vasodilators SNP and Ach.

**Results:**

HCD resulted in a progressive expansion of dWAT and increased expression of pro-inflammatory markers (IL1β and COX-2). Impairments in pressure-induced (PIV) and Ach-induced (endothelium-dependent) vasodilation occurred early, in overweight-metabolically healthy mice. Residual vasodilatory responses were NOS-independent but sensitive to COX inhibition. These changes were associated with reductions in NO and adiponectin bioavailability, and rescued by exogenous adiponectin or hyperinsulinemia. Obese-prediabetic mice continued to exhibit impaired Ach-dependent vasodilation but PIV appeared normalized. This normalization coincided with elevated endogenous adiponectin and insulin levels, and was sensitive to NOS, COX and PI3K, inhibition. In obese-type 2 diabetic mice, both Ach-stimulated and pressure-induced vasodilatory responses were increased through enhanced COX-2-dependent prostaglandin response.

**Conclusions:**

We demonstrate that the development of obesity, metabolic dysfunction and type 2 diabetes, in HCD-fed mice, is accompanied by increased dermal adiposity and associated metaflammation in dWAT. Importantly, these temporal changes are also linked to disease stage-specific dermal microvascular reactivity, which may reflect adaptive mechanisms driven by metaflammation.

## Introduction

With the increased prevalence of obesity and type 2 diabetes, and limited success in preventative approaches, there is an urgent need to better understand and manage the long-term consequences of metabolic disease [1]. Obesity complications include skin disorders that may increase the prevalence of more severe pressure ulcers (PU) [2, 3]. For example, obesity is associated with decreased tensile strength [4] and dermal elasticity in mice [5] and humans [6]. However, an ‘obesity paradox’ has also been reported wherein people with a body mass index (BMI) between 25 and 40 appear to be protected from the development of PU [7]. Indeed, we have recently found that in a murine model of diet-induced obesity, pressure-induced ischaemia and skin lesions are reduced with increasing obesity [8]. This suggests that pressure-induced regulation of cutaneous blood flow may be altered by changes in dermal adiposity. However, this and the underlying mechanisms currently remain unclear. In addition, none of the clinical studies focused on PU incidence have assessed the metabolic status of the obese subjects under investigation. Hence, the impact of increased dermal adiposity per se, or that of the metabolic deregulation that accompanies obesity-linked type 2 diabetes, on vascular fragility of the skin remains unclear.

Mechanistically, numerous features of obesity-associated metabolic deregulation could impact dermal microvascular functionality through local paracrine interactions with expanding adipose tissue. These include obesity-associated impaired metabolic functionality of adipose tissue, altered adipokine production [9] and low-grade chronic inflammation (metaflammation) [9–11]. Some of these have been implicated in perivascular adipose tissue-mediated, endothelial cell dysfunction in arteries and arterioles [12, 13]. Another major causal feature of obesity-linked type 2 diabetes is insulin resistance, which induces endothelial dysfunction in vascular disease via an inadequate production of endothelial NO and endothelin-1 [14, 15]. Among the dermal changes linked to diabetes [1], the disruption of microvascular adjustment to pressure, as revealed by pressure-induced vasodilation (PIV), correlates with increased vascular fragility of the skin [16–19]. Type 2 diabetic patients also exhibit a range of vascular, oxidative stress and inflammatory changes [20] that may affect skin and neurovascular quality [21, 22]. The potential impact of obesity-linked type 2 diabetes on the arterial microenvironment [23] could affect microvascular adjustment to pressure in a context-dependent manner, by changes in adiposity, followed by progressive changes in metabolic dysfunction prior to the establishment of type 2 diabetes.

In this study, we investigate the temporal changes in dermal adiposity, dermal microvascular functionality and in endothelial function during the development of obesity and type 2 diabetes. We hypothesize that remodelling of dermal adipose layer and the development of type 2 diabetes are linked to changes in dermal microvascular reactivity to pressure. Our findings suggest that initially, at the onset of increased adiposity, alterations in neurovascular and endothelial function are associated with altered adipokine production. However, as obesity progresses to pre-diabetic and diabetic states, additional adaptions occur to normalize and then enhance dermal vascular reactivity to pressure. Mechanistically, these adaptive changes involve a shift in key vasodilatory signalling pathways from a NO-dependent to pro-inflammatory COX-2/PG-driven programmes.

## Research design and methods

### Animals

Male C57Bl/6J mice (aged 10 weeks and approximately 25 g from Janvier®, Le Genest-Saint-Isle, France) were acclimated for 1 week prior to start of the study. All animal procedures were carried out in accordance with the principles of French legislation and the ethics committee for animal experimentation at the University of Lyon 1, France. Mice were housed five per cage, maintained at 21 °C, on a 12-h light/dark cycle and at 11 weeks of age were randomly divided into six groups. Mice were placed on either a normal chow diet (control) or an obesogenic hypercaloric diet (HCD) for a further 2, 4 or 12 weeks. The energy-rich diet pellets comprised of 45% Atwater fuel energy (AFE) from fat and 16% AFE from sucrose (total energy 4.5 kcal/g; SDS DIO diet cat# 824127, Essex, UK), and was further supplemented with ad libitum access to sweetened condensed milk (55% simple sugar, 8% fat, 8% protein, w/w, Nestle®) as previously described [8, 24, 25]. Three time points of HCD feeding were identified to represent three distinct stages of disease progression: (a) an early point (2 weeks of HCD, 2HCD) when HCD fed mice first become significantly heavier but fasting glucose remained unaltered, (b) an intermediate point (4 weeks of HCD, 4HCD) when body weight was increased further, but fasting glucose levels were significantly albeit mildly elevated to pre-diabetic levels (see definition below) and (c) a final time point (12 weeks of HCD, 12HCD) when body weight differences were at their greatest and fasting glucose levels were considered diabetic.

### Glucose and insulin tolerance tests

After 2, 4 and 12 weeks of diet, glucose and insulin tolerance tests were performed on 5 h-fasted mice after an intraperitoneal (ip) injection of either glucose (1 g kg^−1^ of body weight) for glucose tolerance test or insulin (0.75 U kg^−1^ of body weight; Lilly, Suresnes, France) for insulin tolerance test. Blood glucose was monitored for 2 h with a glucometer (AccuCheck® Active; Roche, Lyon, France). Mice with baseline fasting blood glucose levels >250 mg/dL were considered to be diabetic, while levels between 200 and 250 mg/dL were defined as prediabetic and <199 mg/dL were normal [26, 27].

### Assessment of dermal microvascular reactivity

For microvascular experiments, animals were anesthetized by ip injection of thiopental sodium (65 mg kg^−1^; Nesdonal, Merial, Lyon, France) and mice, in prone position, were rested in an incubator with controlled dermal temperature and systolic arterial blood pressure as previously described [28]. Any skin lesion on the animal the day of the experiment led to exclusion.

#### Endothelium-dependent and independent vasodilation

Laser Doppler flux (LDF) in response to acetylcholine (Ach) and sodium nitroprusside (SNP) (Saint Quentin Fallavier, France) iontophoresis was measured to assess skin microvascular reactivity on the hairless back of the animals as previously described [19, 28].

#### Pressure-induced vasodilation

Local pressure-induced LDF response was measured as previously described [19, 28]. The pressure probe was placed on hairless skin of the top of the head, and the external pressure was increased progressively at 2.2 Pa s^−1^ through the laser Doppler probe, using a syringe pump.

#### Pharmacological inhibition studies

Stimulated vasodilation was assessed after pretreatment with DMSO (control), wortmannin to inhibit phosphatidylinositol 3-kinase (PI3K) or N^ω^-nitro-L-arginine (LNNA), an NO synthase (NOS) inhibitor. Systolic arterial blood pressure was measured to check the increase in blood pressure caused by LNNA, a specific NOS inhibitor (20 mg kg^−1^; ip) [28]. For wortmannin injection (in 4% DMSO; 16 µg kg^−1^; ip), the protocol was conducted as described above after a resting period of 15 min [29]. Indomethacin (5 mg kg^−1^; i.p.), was used as a non-specific inhibitor of cyclooxygenases (COX) [30, 31] while specific inhibition of inducible COX-2 (Cayman Chemicals, MI, USA) was achieved with SC-58125 (10 mg kg^−1^; i.v.) [32]. The role of insulin and adiponectin in PIV was assessed by either a single ip injection of insulin (0.05 UI 25^−1^ g of mouse body weight; Lilly, Suresnes, France) or a single intradermic injection of adiponectin (50 µg.mL^-1^; Enzo LifeSciences, Farmingdale, NY). Assessment of PIV was conducted 15 min after insulin injection and immediately after adiponectin injection.

At the end of vascular experiments, the animals were euthanized by an overdose of thiopental.

### Plasma biochemistry

Plasma was obtained by cardiac puncture and was analysed by the Centre de Biologie Est (Lyon, France) for lipids (cholesterol/triglyceride, Abbott Laboratories, Abbott Park, IL; free fatty acid, Wako Chemicals, Osaka, Japan), commercial colorimetric ELISA assays were used for determination of plasma insulin (Mercodia, Uppsala, Sweden) and adipokines (leptin/adiponectin, Millipore, St. Charles, MO) according to manufacturer’s protocol.

### Western blotting

Immunoblotting using anti-COX-2 (Cayman Chemicals, #160126) was performed on skin harvested from each group. Pharmacological drugs efficiency was confirmed by immunoblotting using anti-phospho-eNOS-Ser^1177^, anti-phospho-Akt-Ser^473^ (Cell signalling Technology; Danvers, MA) on treated skin harvested at the maximum of PIV.

### Immunohistochemistry and morphometric analysis

Mouse skin specimens were fixed with neutral buffered formalin, dehydrated, embedded in paraffin and cut in 5 μm sections. For the histological analysis, sections were labelled with hematoxylin–eosin–safran. For the immunohistological study, deparaffinized and rehydrated tissue sections were pretreated with citrate buffer (pH = 6.0). Incubation with primary antibodies (anti-F4/80 [Sigma, HPA002274] and anti-IL-1β [Abcam ab9722]) was followed by blocking of endogenous peroxidase activity with 0.5% aqueous H_2_O_2_. After incubation with HRP-conjugated secondary antibodies, sections were revealed with diaminobenzidine, counterstained with Mayer’s hematoxylin and observed using a DM 4000B microscope (Leica) coupled to a colour camera (Digital Camera DXM1200, Nikon). Image acquisition was achieved using Metaview software (Universal Imaging). Measurements of adipocyte number and area (µm^2^) was performed from hematoxylin–eosin–safran-stained histological sections. Histological images (G:x20, 5 pictures/sample, *n* = 4/group) were captured using a wide field optical microscope, then analysed to count the number and the area of each adipocyte. The sequence of image analysis steps was automatically implemented by Adiposoft 1.14 in Manual Mode. This software was calibrated based on the magnification used during image acquisition. The number and area (in µm^2^) occupied by each adipocyte was automatically calculated by Adiposoft software.

### Statistical analysis

Investigators were not blinded to treatment and data analysis. Statistical analysis was done using GraphPad Prism software (GraphPad Software, Inc., San Diego, CA). The data is reported as mean ± standard error of the mean (SEM). Shapiro and Wilks goodness-of-fit test was used to test for normal distribution. Variance was assessed for each dependent variable measured. Statistically significant differences were calculated by one-way analysis of variance (followed by Duncan’s post-test) or unpaired Student’s *t*-test; *p* < 0.05 was considered statistically significant. Sample size for each test was based on previous studies using mice models.

## Results

### Temporal phenotyping of diet-induced obesity reveals specific stages of metabolic disease development and acute changes in adiponectin levels

**Fig. 1:**
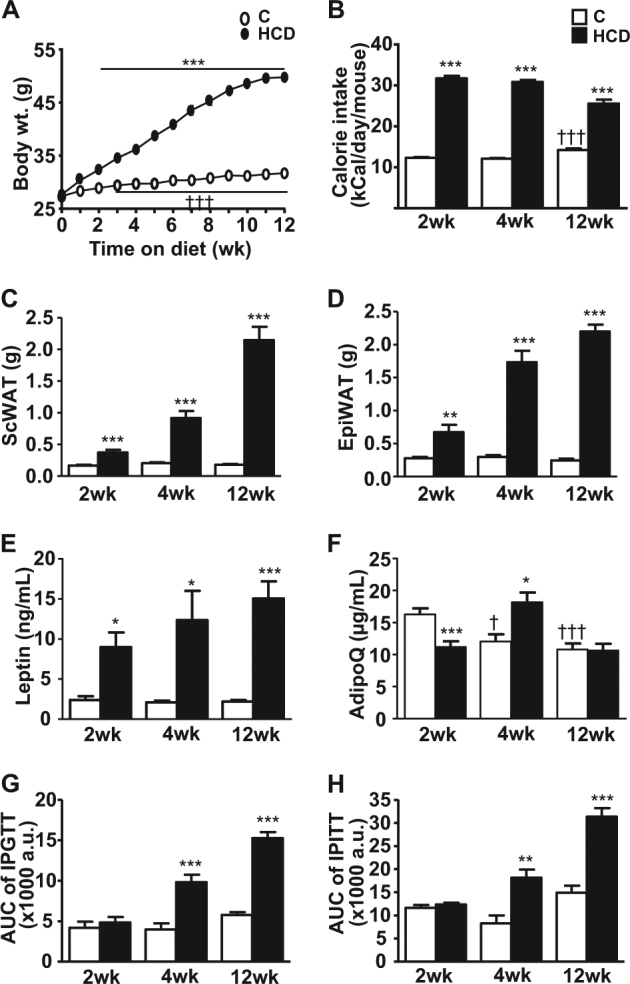
Effect of HCD on the development of obesity and metabolic syndrome. Mice were fed either hypercaloric diet (filled circles and bars) or standard chow (open circles and bars) for 2, 4 or 12 weeks. **a** Body weight of mice during the 12-week feeding programme. **b** Calorie intake of control and HCD fed mice. **c** Subcutaneous white adipose tissue (ScWAT) weights. **d** Epididymal white adipose tissue (EpiWAT) weights. **e** Circulating leptin levels. **f** Circulating adiponectin (AdipoQ) levels. **g** Area under the curve (AUC) of glucose levels during glucose tolerance tests (IP-GTT) and **h** Insulin tolerance tests (IP-ITT). See also Supplemental Fig. 1. Data represents mean ± SEM (*n* = 10 in each group or *n* = 50 in each group for body weight) *p < 0.05, **p < 0.01, ***p < 0.001 vs. age-matched control diet fed mice. ^†^*p* < 0.05, ^††^*p* < 0.01, ^†††^*p* < 0.001 vs. 2-week control diet fed mice

Body weight was significantly elevated early at 2 weeks after HCD feeding and continued to increase with duration on HCD diet compared to age-matched control mice (Fig. 1a). This was accompanied by greater caloric intake (Fig. 1b) and progressively greater adiposity in inguinal subcutaneous (Fig. 1c) and epididymal (Fig. 1d) adipose depots. This coincided with an early and progressive increase in adiposity-related adipokine, leptin (Fig. 1e). However, circulating triglycerides and non-esterified fatty acids did not change until 12 weeks after the start of HCD feeding (Table S1).

There was an incremental development of type 2 diabetes in this model. After 2 weeks of HCD, plasma adiponectin levels (Fig. 1f) were lower but mice remained euglycemic, and no differences were observed in plasma insulin levels (Table S1), glucose tolerance (Fig. 1g and Fig. S1A) or insulin tolerance (Fig. 1h and Fig. S1B). Hence, this 2-HCD group represented a model of ‘overweight-metabolically healthy’ mice albeit with reduced adiponectin. After 4 weeks, 4-HCD mice were obese and had normalized adiponectin levels (Fig. 1f) and were very mildly hyperinsulinemic and hyperglycemic (Table S1). Both glucose- (Fig. 1g and Fig. S1C) and insulin- (Fig. 1h and Fig. S1D) tolerance were also mildly impaired compared to age-matched control diet fed mice. Hence, this 4-HCD group represented a model of ‘obese-prediabetic’ mice. Lastly, 12-HCD mice exhibited the greatest levels of obesity, hyperglycaemia, hyperinsulinemia and hyperlipidaemia (Table S1) together with impaired glucose- and insulin-tolerance. (Fig. 1g, h and Fig. S1E-F). Hence, these mice exhibited an established disease stage characteristic of ‘obesity and type 2 diabetes’.

It is noteworthy that control diet fed lean mice also exhibited time-dependent alterations in their metabolic profiles that became significant after 12 weeks. The changes included a small but significant increase body weight (Fig. 1a, Table S1) and food intake (Fig. 1b), but did not translate into significantly larger WAT depots (Fig. 1c, d) nor increased plasma leptin (Fig. 1e). The oldest lean mice remained tolerant to both glucose (Fig. 1g) and insulin (Fig. 1h). However, plasma adiponectin levels did progressively decline (Fig. 1f) and mild fasting hyperglycaemia was observed by 12 weeks (Table S1).

### Remodelling of dWAT accompanies the development of HCD-induced obesity

**Fig. 2:**
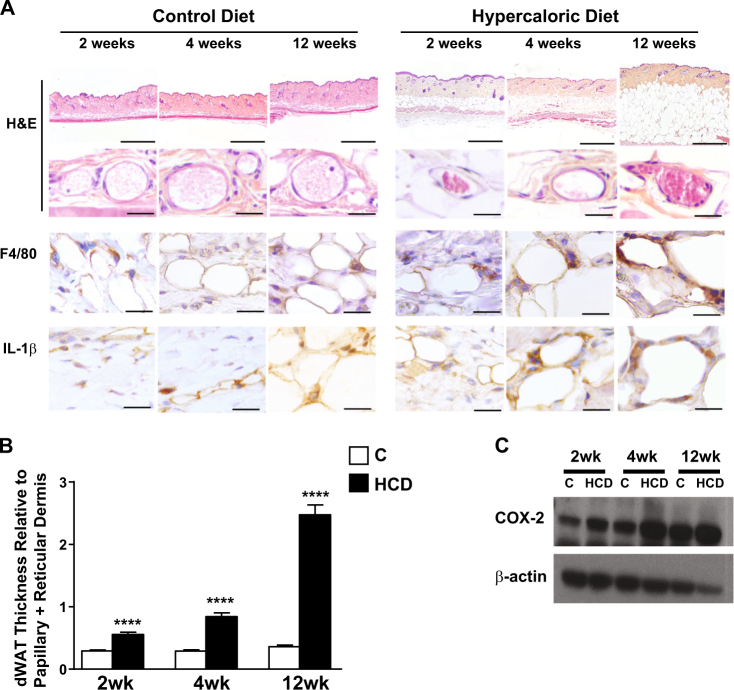
Effect of HCD on dermal adipose remodeling and inflammation. **a** Representative photomicrographs of immunohistochemical sections from indicated groups following staining with hematoxylin and eosin, F4/80 or anti-IL1-beta. (Scale bars: 500 µm (low mag) and 25 µm (high mag).) **b** Thickness of dermal adipose tissue was determined relative to the combined papillary dermis and reticular dermis thickness. For each of five mice, 10 randomly selected measurements were calculated from histological images and measured using image J. Data represents mean ± SEM (*n* = 5 in each group) *p < 0.05, **p < 0.01, ***p < 0.001 vs. age-matched control diet fed mice. **c** Effects of age and HCD on COX-2 expression in mouse skin

In HCD-fed mice, the dermal adipose layer [33] was already expanded at the earliest time point (Fig. 2a, b). The dWAT occupied greater area with time on HCD (Fig. 2a, b and Fig. S2A) and was accompanied by a progressive increase in both adipocyte hyperplasia and hypertrophy compared to control diet fed mice (Fig. 2a and Fig. S2B). Immunohistochemical staining for macrophages (anti-F4/80) and pro-inflammatory cytokines (anti-IL-1β) revealed a progressive increase in macrophage infiltration after 4 weeks (Fig. 2a). In 12-HCD mice, there was a noticeable clustering of pro-inflammatory (M1-like) macrophages in ‘crown-like’ structures. In skin biopsies, the protein levels of inflammation-associated COX-2 also progressively increased with increased dermal adiposity (Fig. 2c and Fig. S2C). However, there was no change in the structure of the dermal vessels in HCD and control mice (Fig. 2a).

### Dermal microvascular functionality is altered in a disease-stage-specific manner

**Fig. 3:**
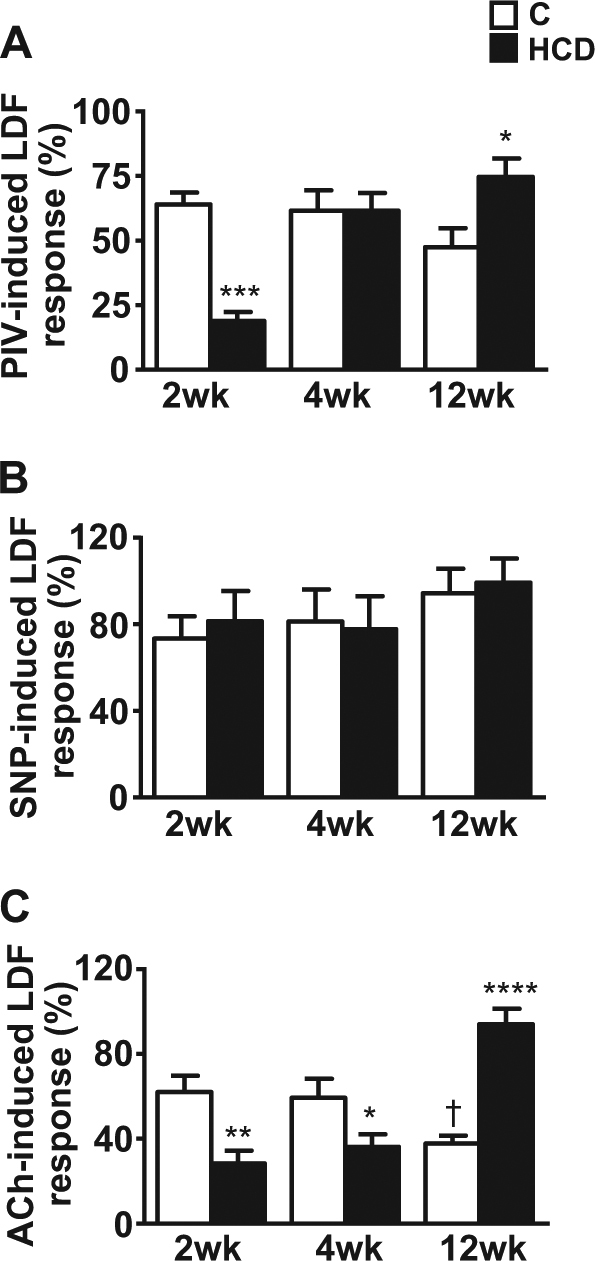
Time-dependent effects of HCD on vascular reactivity. Maximal percent increase in skin laser Doppler flowmetry (LDF) was determined in response to **a** mild pressure, **b** iontophoretic delivery of SNP and **c** Ach. Data represents mean ± SEM (*n* = 10 in each group); *p < 0.05, **p < 0.01, ***p < 0.001, ****p < 0.0001 vs. age-matched control diet fed mice; ^†^*p* < 0.05 vs. 2-week control diet fed mice

We next investigated the temporal changes in dermal blood flow. Basal LDF increased with the duration on HCD diet and when corrected for systemic arterial blood pressure (SABP), the vascular conductance (VC) remained significantly increased in 12-HCD mice (Fig. S3). In 2-HCD mice, the LDF response to local pressure application was significantly reduced compared to age-matched control lean mice (Fig. 3a). However, this PIV response appeared normalized in 4-HCD mice, and was significantly increased in 12-HCD mice compared to age-matched lean controls. In contrast, maximal SNP-stimulated (endothelium-independent) vasodilation remained similar between HCD and control groups (Fig. 3b) in all groups, suggesting dermal smooth muscle cell relaxation capacity is not altered during the development of obesity and type 2 diabetes. However, Ach-stimulated (endothelium-dependent) vasodilation was impaired at an early disease-stage, in both 2-HCD and 4-HCD mice (Fig. 3c). Remarkably, 12-HCD mice exhibited a greater vasodilatory response to Ach, which was due in part, to a reduction in Ach-stimulated vasodilation in control lean mice (Fig. 3c) that may reveal temporal endothelial dysfunction similar to 2-HCD mice. However, cholesterol levels remained in the normal range in all groups excluding any confounding effect of hypercholesterolaemia linked to obesity/type 2 diabetes on microvascular changes.

### Impaired PIV and endothelial dysfunction in overweight-metabolically healthy mice is associated with loss of NO and adiponectin bioavailability

**Fig. 4:**
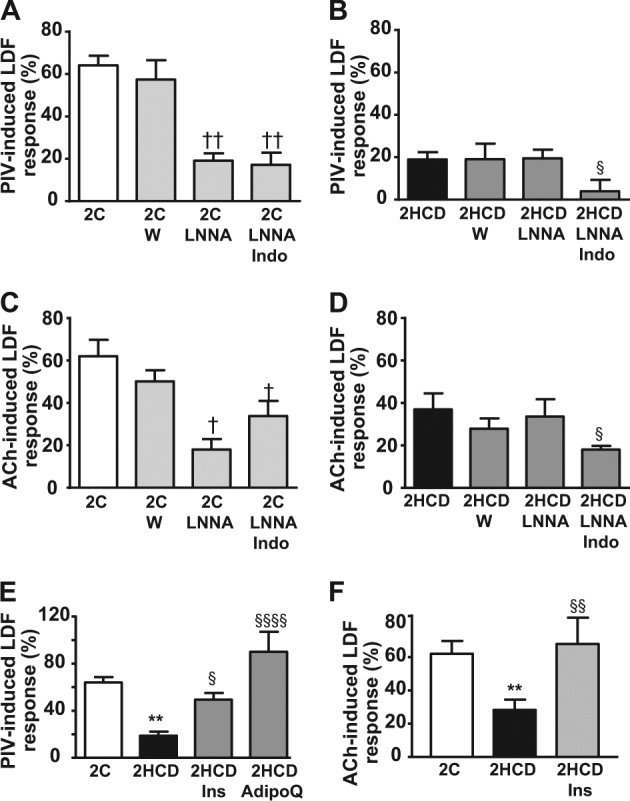
Mechanisms involved in impaired PIV and Ach responsiveness in overweight metabolically healthy mice. Mice were fed either a standard chow (C; white or light grey bars) or hypercaloric diet (HCD; black or dark grey bars) for 2 weeks. Effects of selected pharmacological inhibitors were determined on microvascular response to local pressure application (**a**, **b**) or Ach stimulation (**c, d**). Inhibitors included PI3-kinase inhibitor, wortmannin (W; *n* = 10), nitric oxide synthase inhibitor, L-NG-nitro-L-arginine (LNNA; *n* = 10) and LNNA + anti-inflammatory COX-1/2 inhibitor, indomethacin (LNNA + Indo; *n* = 5). **e** Effects in vivo administration of insulin (Ins; *n* = 10) and adiponectin (AdipoQ; *n* = 9) on impaired PIV response in 2-week-HCD fed mice. **f** Effects in vivo administration of insulin (Ins; *n* = 6) on impaired Ach-induced response in 2-week-HCD fed mice. Data represents mean ± SEM. ^†^*p* < 0.05, ^††^*p* < 0.01 vs. 2-week-C diet fed mice; *p < 0.05, **p < 0.01 vs. age-matched control diet fed mice; and ^§^*p* < 0.05, ^§§^*p* < 0.001, ^§§§§^*p* < 0.00001 vs. 2-week hypercaloric diet fed mice

We next investigated the mechanisms responsible for the early impairment in PIV and Ach-stimulated endothelial function in overweight-metabolically healthy (2-HCD) mice. Unlike its effects in control lean mice, the NOS inhibitor, LNNA had no effect on either PIV- (Fig. 4a, b) or Ach- (Fig. 4c, d) stimulated vasodilation in 2-HCD mice. This suggests that NOS-dependent vasodilation is lost. The PI3K inhibitor, wortmannin, had no effect on both vasodilatory tests in both diet groups (Fig. 4a–d). However, western blotting for phosphoactive-AKT and phosphoactive-eNOS confirmed that these inhibitors were effective at the molecular level (Fig. S4). Additionally, treatment with indomethacin, a COX-1/2 inhibitor, demonstrated that residual vascular functionality in 2-HCD mice, was sensitive to prostaglandin-dependent signals (Fig. 4a–d).

Since the additional requirement for prostaglandin-dependent vasodilation coincided with a decrease in the anti-inflammatory adipokine, adiponectin (Fig. 1f), we hypothesized that reduced PIV in 2-HCD mice was due to reduced adiponectin bioavailability. This was supported by the complete reversal of impaired PIV response following a single injection of adiponectin in 2-HCD mice (Fig. 4e). As adiponectin also has insulin-sensitizing properties [34], 2-HCD mice were next pre-treated with insulin supplementation prior to PIV determination. This pre-treatment raised plasma insulin levels to 9.37 ± 1.7 µg/L (*p* < 0.001) and did not alter basal LDF (data not shown). However, elevating insulin levels reversed the impaired PIV and Ach-stimulated vasodilation in 2-HCD mice (Fig. 4e, f).

### Normalization of PIV in obese-prediabetic mice requires adaptive PI3K-dependent NO signals

**Fig. 5:**
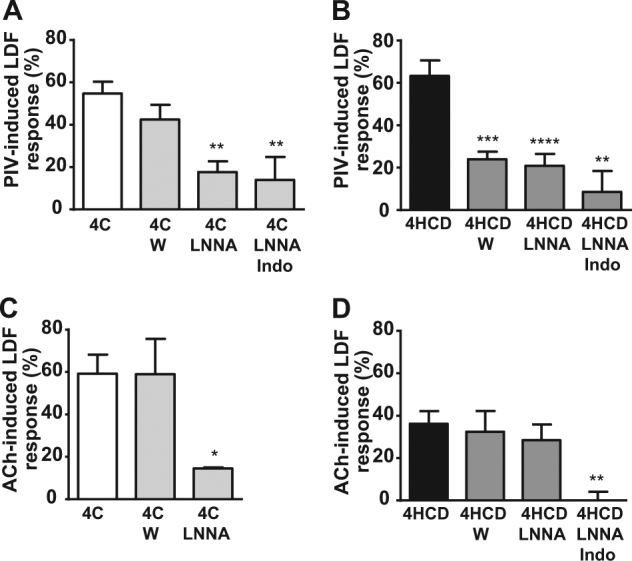
Mechanisms involved in normalized PIV and impaired Ach responsiveness in obese pre-diabetic mice. Mice were fed either hypercaloric diet (HCD; black or dark grey bars) or standard chow (C; white or light grey bars) for 4 weeks. Effects of selected pharmacological inhibitors were determined on microvascular response to local pressure application (**a**, **b)** or Ach stimulation (**c, d**). Inhibitors included PI3-kinase inhibitor, wortmannin (W; *n* = 7), nitric oxide synthase inhibitor, L-NG-nitro-L-arginine (LNNA; *n* = 10) and LNNA + anti-inflammatory COX-1/2 inhibitor, indomethacin (LNNA + Indo; *n* = 5). Data represents mean ± SEM; *p < 0.05, **p < 0.01, ***p < 0.001, ****p < 0.0001 vs. age-matched mice fed the same diet but not treated with any inhibitor

Since obese-prediabetic (4-HCD) mice had elevated insulin (Table S1**)** and normalized adiponectin levels (Fig. 1f), and PIV responses had become similar to age-matched lean mice (Fig. 3a), we explored the possibility that hyperinsulinemia-driven insulin signalling was involved in the adaptive mechanism to normalize PIV. To explore the requirement for a functional insulin receptor/PI3K/AKT/NOS signalling pathway, we repeated PIV and Ach-stimulated tests after administering pathway selective inhibitors in 4-HCD mice. Here, PIV was sensitive to NOS inhibition by LNNA and to the PI3K inhibitor, wortmannin (Fig. 5b). In contrast, control mice remained insensitive to wortmannin (Fig. 5a). This indicates the recruitment of a PI3K-dependent NOS pathway in PIV response of 4-HCD mice, while a PI3K-independent NOS pathway remained functional in control lean mice. Interestingly, wortmannin and NOS sensitivities were not observed in Ach-stimulated vasodilation (Fig. 5c,d) in 4-HCD mice (Fig. 5d). However, reactivity to Ach remained indomethacin-sensitive (COX-dependent) in 4-HCD mice, as in 2-HCD.

### Enhanced PIV and acetylcholine-stimulated vasodilation in obese-diabetic mice requires COX-2

**Fig. 6:**
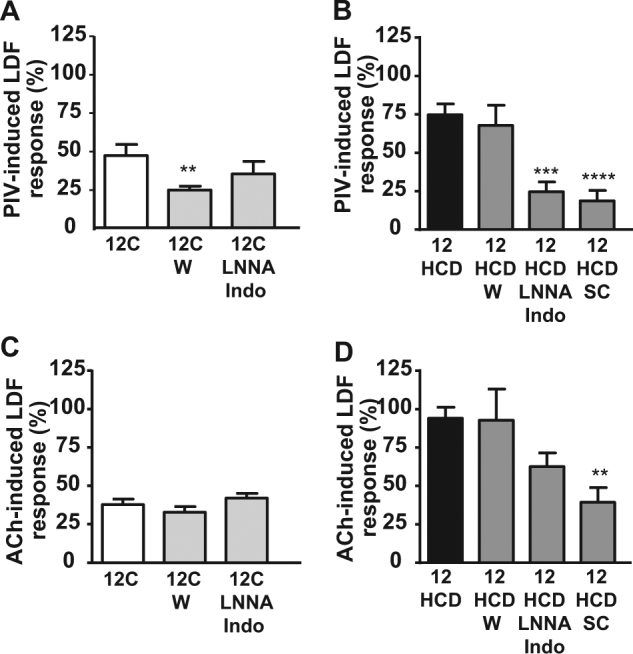
Mechanisms involved in enhanced PIV and ACh responsiveness in obese diabetic mice. Mice were fed either hypercaloric diet (HCD; black or dark grey bars) or standard chow (C; white or light grey bars) for 12 weeks. Effects of selected pharmacological inhibitors were determined on microvascular response to local pressure application (**a**, **b**) or Ach stimulation (**c**, **d**). Inhibitors included PI3-kinase inhibitor, wortmannin (W; *n* = 10), nitric oxide synthase inhibitor, L-NG-nitro-L-arginine (LNNA) and anti-inflammatory COX-1/2 inhibitor, indomethacin (Indo; *n* = 7) and SC5812 (SC; *n* = 7). Data represents mean ± SEM; *p < 0.05, **p < 0.01, ***p < 0.001, ****p < 0.0001 vs. age-matched mice fed the same diet but not treated with any inhibitor

In obese-diabetic (12-HCD) mice, both PIV and Ach-stimulated vasodilation were significantly enhanced compared to age-matched lean controls (Fig. 6a–d). Although plasma insulin levels had elevated further (Table S1), adiponectin levels were now similar to lean mice (Fig. 1f). Here, PIV and Ach-stimulated responses were no longer sensitive to PI3K inhibition by wortmannin in 12-HCD (Fig. 6b). Nonetheless, enhanced PIV and Ach were sensitive to combined inhibition of NOS and COX-1/2 inhibition (LNNA + Indo). Moreover, specific inhibition of COX-2 by SC58125 alone resulted in the same magnitude of inhibition as that achieved by dual LNNA + Indo (Fig. 6b, d). This suggests a major role for COX-2 in PIV and Ach responses in 12-HCD mice.

## Discussion

Alterations in skin fragility and neurovascular function are commonly associated with complications of diabetes and include increased PU risk. However, the ability of overweight and obese subjects to regulate protective neurovascular mechanisms, such as PIV, is not known. Indeed, the impact of increased dermal adiposity on dermal blood flow and microvascular functionality is unclear. This systematic investigation of disease ontogeny in a model of diet-induced obesity and type 2 diabetes has identified temporal changes in dermal tissue remodelling and microvascular functionality that accompany increased adiposity, development of metabolic deregulation and metaflammation. We confirm that HCD fed mice develop obesity-associated type 2 diabetes within 12 weeks (12-HCD) but this is preceded by at least two distinct disease stages: ‘overweight-metabolically healthy’ (2-HCD) and ‘obese-prediabetics’ (4-HCD). Importantly, we show for the first time, that (1) dysfunction in dermal microcirculation can be detected early during the onset of diet-induced adiposity, when dermal adipose tissue is increased but whole-body metabolic status remains minimally altered; (2) progression to obesity-related type 2 diabetes is accompanied by enhanced adaptive dermal vasodilatory responses and (3) the adaptive microvascular responsiveness involves a sequential switch from NO-dependent programme to pro-inflammatory Cox2/PG programmes.

Our finding that dysfunction in dermal microvasculature is an early feature of diet-induced adiposity in overweight-metabolically healthy mice, is consistent with clinical and pre-clinical studies, which demonstrate decreased reactivity in other vascular beds following a single high-fat meal [35–37]. Our observation of impaired responsiveness to two stimuli (pressure and Ach) suggests that the primary defect at this stage may be dysfunction in endothelial-derived NO production. Indeed, reduced NO bioavailability has been associated with acute hyperglycaemia-mediated oxidative stress [38, 39]. However, unlike most studies of endothelial dysfunction, our vasodilatory tests were performed in-situ—in direct contact with innervated dWAT. Here, we demonstrate that neurovascular and endothelial dysfunction can be impacted early by altered adipokine production from expanding dWAT. We show that impaired PIV in overweight-metabolically healthy mice is linked to decreased bioavailability of the vasoactive adipokine, adiponectin. Although adipose expression of adiponectin is reduced in obesity [9, 10], in our model we did not see a sequential decrease of adiponection levels in HCD fed mice. The transient elevation in adiponectin levels (in 4HCD mice) is reminiscent of adiponectin resistance [9]. Interestingly, impaired adiponectin signalling has been reported at both receptor and post-receptor levels. For example, adiponectin receptor 1 has been shown to be expressed on adipose tissue-resident Tregs, and this expression is negatively correlated with epididymal fat mass even in the absence of significant changes in serum adiponectin [40]. Although dWAT-Tregs have not been investigated, it is possible that in our model, increased adiposity also associates with changes in local immunoregulation via reduced adiponectin sensitivity of dWAT-resident Tregs. Nonetheless, our study extends the understanding of adiponectin involvement in PIV in the context of obesity-linked endothelial dysfunction—previously reported only in major blood vessels surrounded by perivascular adipose tissue [13, 41–43]. Importantly, our finding that a single application of either adiponectin or insulin can restore PIV in these overweight mice supports the positive involvement of these metabolic factors in dermal neurovascular functionality and may offer new diagnostic and/or therapeutic options for PU prevention in overweight individuals.

As increased adiposity progresses to obesity-related metaflammation and type 2 diabetes, we observed enhanced basal blood flow and increased microvasodilatory responses in the dermis. This suggests that unlike the clear pathogenic effects of type 1 diabetes on PIV [16, 19], obesity can drive beneficial neurovascular adaptation in the dermis even in the context of developing type 2 diabetes. Hence, the presence of adiposity has a key role in maintaining and augmenting the vasodilator capacity of dermal microvessels. Moreover, these observations may explain our previous finding that HCD fed obese-diabetic mice become less prone to dermal ischaemia and skin lesions in response to acute skin compression [8].

Mechanistically, a normalized PIV response in obese-prediabetic mice (4-HCD) is not driven by parallel improvements in Ach-induced vasodilation. Discordant responses (between PIV and Ach) have previously been reported [17, 44] and in our model, the adaptive PIV response is likely mediated by the combined action of mild hyperinsulinemia and improvements in adiponectin bioavailability, which enlist PI3K- and NOS-dependent vasodilation. It is noteworthy that unlike Ach-stimulated vasodilation, PIV requires the additional release of vasoactive neuromodulators such as CGRP, which act via G-protein receptors to stimulate endothelial-NO production [45]. Although, details of CGRP-mediated signal transduction remain unclear, it has been reported to activate AKT in a wortmannin-sensitive manner [46] and synergize with inflammatory signals [47, 48]. Moreover, direct interactions have been reported between G-protein receptors [49, 50] and tyrosine kinase receptors (e.g. insulin receptor) making it tempting to speculate that mild hyperinsulinemia with insulin-sensitizing adiponectin (in obese-prediabetic mice) is sufficient to activate a vasoactive pathway involving CGRP, IR-TK, PI3K, AKT and eNOS to promote normalization PIV response. Future studies should address the putative participation of these pathways in regulating PI3K-dependent NOS production during PIV.

Obese-diabetic mice (12-HCD) exhibit all the hallmarks of type 2 diabetes and the metabolic syndrome. However, despite these features, microvascular functionality in response to both pressure and Ach is increased. Although this appears to contrast with reports linking macrovascular endothelial dysfunction to obesity, insulin resistance and type 2 diabetes [20, 21, 51, 52], it is noteworthy that the latter alterations are described for large and medium arteries and are based on different in vivo techniques and stimuli such as flow-mediated dilation (FMD), which activate alternative pathways to trigger vasodilation [53, 54]. Mechanistically, the enhanced adaptive vasodilatory responses in 12-HCD mice are PI3K-independent and consistent with established endothelial insulin resistance. Instead, the vasoactive mediators appear to require Cox-2 activity, which generates the vasodilators PGI2 and PGE2 [55]. This is supported by reports showing rescue of coronary endothelial function by COX-2 overexpression in obese-insulin resistant mice [56, 57]. In addition, the increased abundance of adipocytes and pro-inflammatory (M1-like) macrophages are likely contributors to this adaptive vascular response [58]. They not only promote local cytokine-stimulated vasodilator production but are additional sources of COX-2 activity that collectively may be sufficient to overcome endothelial cell-specific dysfunction. Indeed, perivascular adipose tissue surrounding the human saphenous vein has been shown to attenuate noradrenalin-induced contraction by releasing PGE2 and PGI2 [58]. Therefore, similar adaptive mechanisms may exist to compensate for and maintain functionality in dermal microvascular beds that lie in close proximity to increasing adipose tissue during the progression of obesity and diabetes.

Finally, it is noteworthy that by the end of the 12-week study, lean control diet fed mice exhibited a number of age-related changes that were similar to those observed in mice fed HCD for 2 weeks. This included increase in body weight (Fig. 1a, Table S1), food intake (Fig. 1b), adipocyte hyperplasia and hypertrophy (Fig. S2B), decreased plasma adiponectin (Fig. 1f) and mild fasting hyperglycaemia (Table S1). However, unlike the 2HCD group, these 23-week-old lean mice only exhibited impairment in Ach-stimulated vasodilation with a trend towards decreased PIV. Although residual vasodilatory responses were no longer NO-dependent in both groups of mice, there were some differences in the mechanisms involved in mediating residual vasodilation. Firstly, residual Ach-dependent vasodilation did not gain sensitivity to either COX1/2 or wortmannin inhibition as seen in 2HCD mice. This suggests the involvement of additional age-related mechanisms, e.g. endothelium-derived hyperpolarizing factor (EDHF), can contribute to maintain relaxation of vascular smooth muscle cells when NOS and COX are deficient to produce NO and vasodilatory prostanoids [28]. However, PIV seems to be partly compensated for by the recruitment of PI3K-dependent (wortmannin-sensitive) signals. Taken together, this data further supports the notion that distinct adaptive mechanisms can be recruited to maintain PIV in the face of impaired endothelial function.

In summary, we have demonstrated that increased dermal adiposity, associated metaflammation and metabolic dysfunction drive disease stage-dependent adaptations in dermal microvascular reactivity. The involvement of distinct adaptive vasodilatory signals may prove useful to inform better diagnostics to assess PU risk in overweight and obese subjects with or without altered metabolic profiles.

## Electronic supplementary material

Supplemental Table and Figures
