# Supplementary material for: Inflammation-linked adaptations in dermal microvascular reactivity accompany the development of obesity and type 2 diabetes
Source: Int J Obes (Lond). 2018 Jul 13;43(3):556–66. doi: 10.1038/s41366-018-0148-4 (PMC6223541; doi:10.1038/s41366-018-0148-4)
Supplement: Supplementary file 1 — Supplemental Table and Figures [file 41366_2018_148_MOESM1_ESM.docx]

Nguyen-Tu et al. **Inflammation-Linked Adaptations In Dermal Microvascular Reactivity Accompany The Development of Obesity And Type 2 Diabetes**

**SUPPLEMENTARY TABLES & FIGURES**

**Supplemental Table 1: Metabolic parameters in plasma of control and HCD fed mice.**

Hypercalorific diet (HCD)-induced significant differences compared to age-matched control diet (C) fed mice are indicated by *(p<0.05) or **(p<0.001). *n* = 6–10 per group. Data is reproduced with permission from Nguyen-Tu et al. {Nguyen-Tu, 2013 #936}. Additional age-related differences (compared to 2C group) are indicated here by † (p<0.05) and †† (p<0.001).

|  | **2C** | **2HCD** | **4C** | **4HCD** | **12C** | **12HCD** |
| --- | --- | --- | --- | --- | --- | --- |
| **Body weight (g)** | 28.8±0.2 | 31.7±0.4** | 29.5±0.2 | 35.4±0.5** | 31.6±0.3†† | 51.0±0.4** |
| **Fasting Glucose**  **(mg/dL)** | 154±6 | 177±11 | 161±7 | 198±16** | 179±6† | 297±11** |
| **Insulin**  **(µg/L)** | 1.06±0.29 | 1.35±0.15 | 1.10±0.30 | 2.41±0.41* | 1.24±0.26 | 3.94±1.18* |
| **Total Cholesterol (mmol/L)** | 2.19±0.12 | 2.92±0.10* | 2.37±0.13 | 3.02±0.29 | 1.79±0.42 | 2.77±0.07** |
| **Triglycerides**  **(mmol/L)** | 0.75±0.11 | 0.61±0.05 | 0.70±0.07 | 0.70±0.07 | 0.47±0.07 | 0.83±0.13* |
| **Nonesterified fatty acid**  **(µmol/L)** | 268±80 | 178±19 | 242±29 | 293±40 | 137±24 | 233±17** |

**Supplemental Figure 1. Effects of duration of HCD feeding on glucose and insulin tolerance.**

Blood glucose response to ip injection of **(A, C, E)** glucose (1 g.kg^-1^) or **(B, D, F)** insulin (0.75 U.kg^-1^) in HFD fed (black circles) mice compared and control diet fed (open circles) mice.

Mice were fed study diets for **(A and B)** 2 weeks, **(C and D)** 4 weeks or **(E and F)** 12 weeks.

Mean±SEM (*n=10* in each group*)*; **p<0.01 and***p<0.001 vs Control diet fed mice.


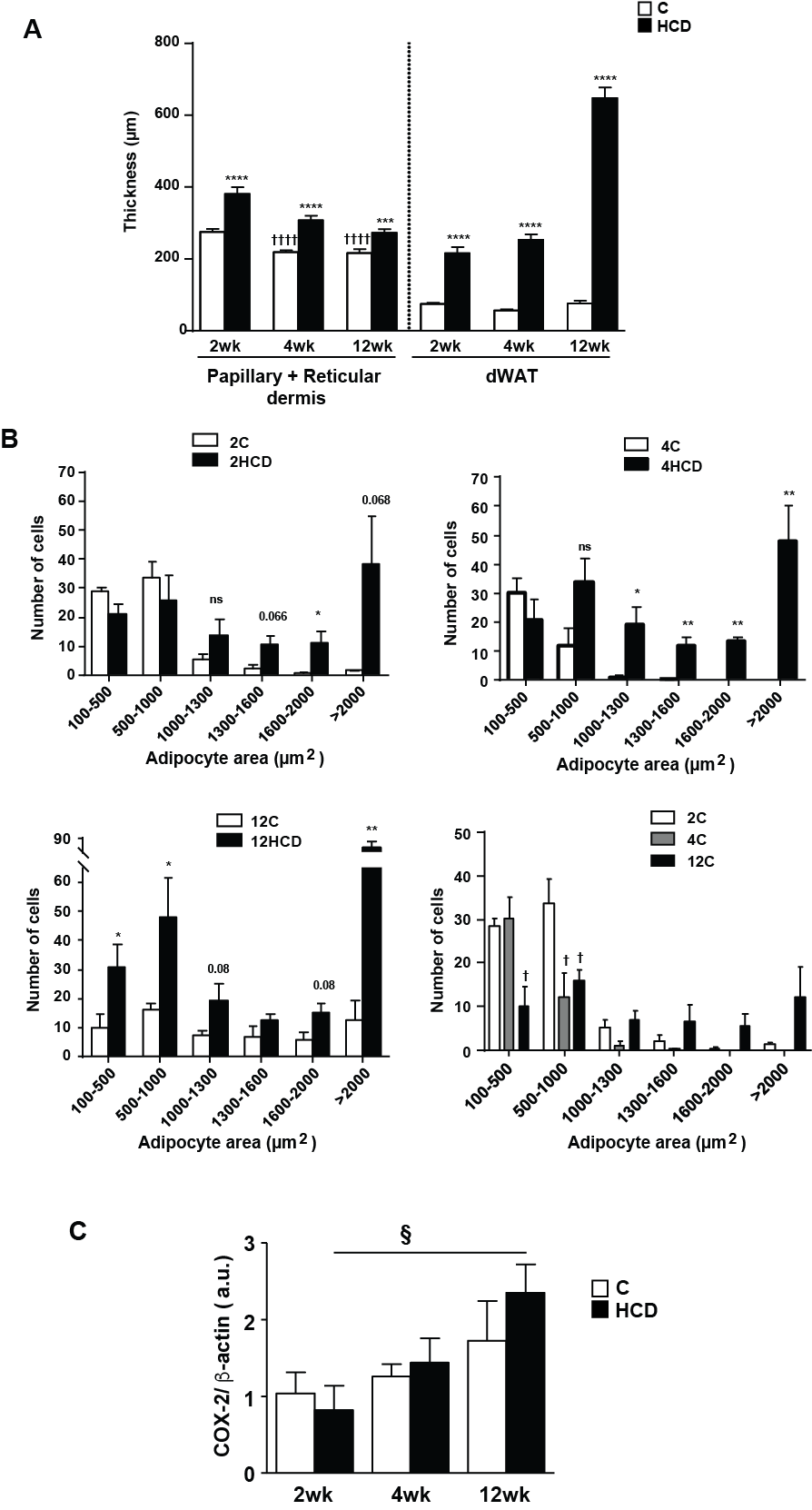


**Supplemental Figure 2: Time dependent effects of HCD feeding on dermal adipose tissue morphometry and quantified Cox-2 expression.**

Mice were fed either hypercaloric diet (HCD; black or dark grey bars) or standard chow (C; white bar) for 2, 4 or 12 weeks. **(A)** Thickness of papillary dermis plus reticular dermis, and dermal white adipose tissue (dWAT) were quantified from 10 randomly selected measurements on each of 5 mice. **(B)** Adipocyte cross section area and number was quantified using adiposoft plugin in ImageJ. **(C)** COX-2 protein expression in mouse skin was determined by western blotting and signal densities quantified. Data represents Mean±SEM and statistical significance is indicated by * p<0.05, ** p<0.01, *** p<0.001, **** p<0.0001 versus age-matched control diet fed mice; † p<0.05 and †††† p<0.0001 versus 2wk control diet fed mice; § p<0.05, HCD group versus C diet fed mice.


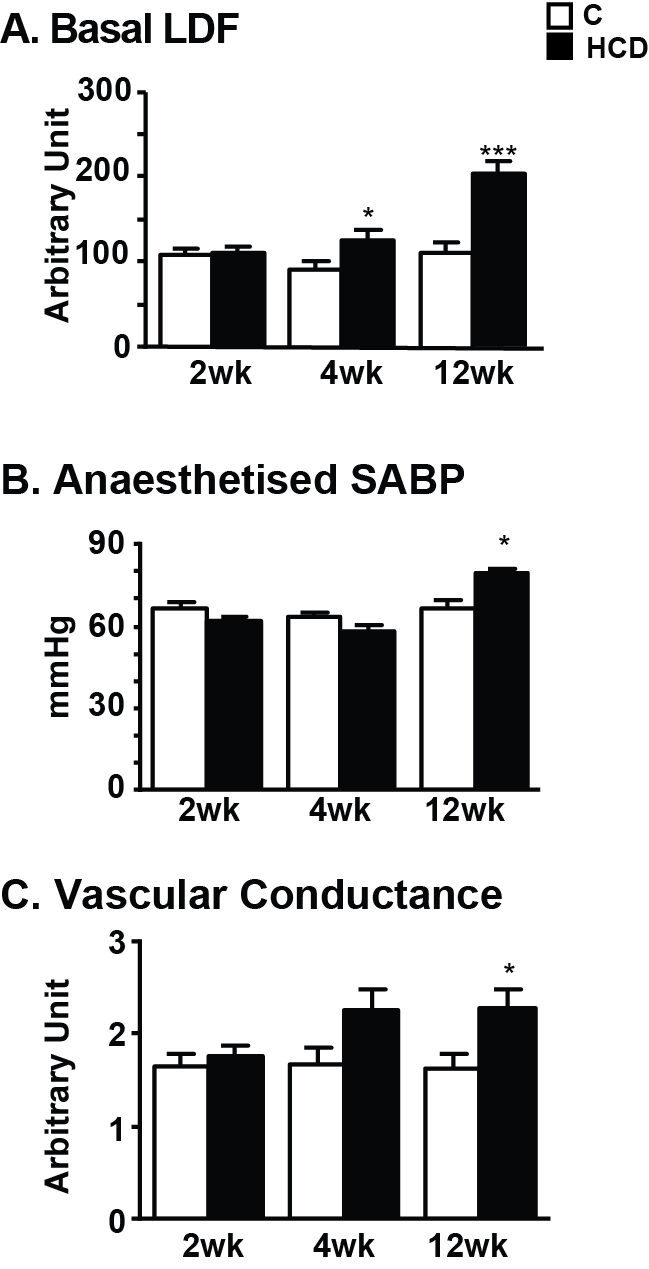


**Supplemental Figure 3: Effect of duration of HCD feeding on basal laser Doppler flow.**

Mice were fed either hypercaloric diet (HCD; black bars) or standard chow (C; white bars) for 2, 4 or 12 weeks. **A.** Basal Laser Doppler Flow (LDF). **B.** Anesthetized systemic arterial blood pressure **C.** Vascular conductance (LDF corrected for SABP). *p<0,05; ***p<0,01 vs respective controls


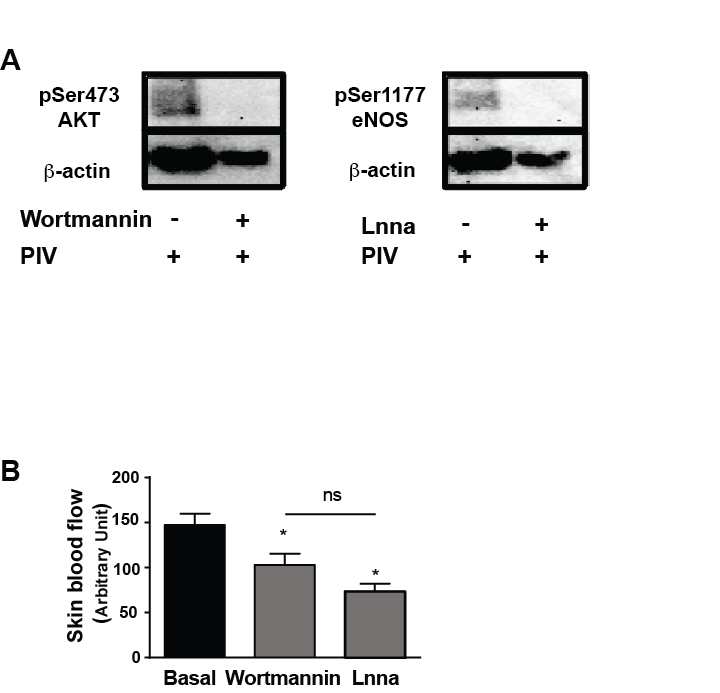


**Supplemental Figure 4: Effects of pharmacological inhibitors on signaling proteins and basal blood flow**

Wortmannin and Lnna were injected in vivo prior to pressure application. **(A)** Representative western blot of p-eNOS, and p-Akt in skin stimulated with PIV and treated with or without inhibitor. **(B)** Effect of wortmannin and LNNA inhibition on basal blood flow in 4-HCD. Mean±SEM. (*n=10* in each group; ***p<0.001 vs control).
